# A numerical study of the effect of varied blood pressure on the stability of carotid atherosclerotic plaque

**DOI:** 10.1186/1475-925X-13-152

**Published:** 2014-11-20

**Authors:** Huahua Xiong, Xin Liu, Xiaohong Tian, Lina Pu, Heye Zhang, Minhua Lu, Wenhua Huang, Yuan-Ting Zhang

**Affiliations:** Department of Ultrasound, The Second People’s Hospital of Shenzhen, Shenzhen, China; Key Lab of Health Informatics of Chinese Academy of Sciences, Shenzhen, China; Institute of Biomedical and Health Engineering, Shenzhen Institutes of Advanced Technology, Shenzhen, China; Institute of Clinical Anatomy, Southern Medical University, Guangzhou, China; Joint Research Centre for Biomedical Engineering, The Chinese University of Hong Kong, Shatin, Hong Kong; Cardiac Electrocardiogram Room, The Second Peoples’ Hospital of Shenzhen, Shenzhen, 518029 China; Department of Biomedical Engineering, School of Medicine, Shenzhen University, Shenzhen, China; University Town, 1068 Xueyuan Ave., Xili, Nanshan district, Shenzhen, Guangdong 518055 China

**Keywords:** Atherosclerotic plaque vulnerability, Stress distribution, Blood pressure, Computational mechanical analysis

## Abstract

**Background:**

Blood pressure (BP) is associated with early atherosclerosis and plaque rupture because the BP variability can significantly affect the blood flow velocity and shear stress over the plaque. However, the mechanical response of BP variability to the plaque remains unclear. Therefore, we investigated the correlation between different maximum systolic blood pressure (SBP) and the stress distribution on plaque, as well as the stress over the plaque and blood velocity around the plaque using different BP variations, which are the BP variability in different phases during one cardiac cycle and beat-to-beat BP variability.

**Method:**

We established a two-dimensional artery model with stenosis at the degree of 62.5%. Eight combinations of pulsatile pressure gradients between the inflow and outflow were implemented at the model. Three levels of fibrous cap thickness were taken into consideration to investigate the additional effect on the BP variability. Wall shear stress and stress/strain distribution over the plaque were derived as well as the oscillation shear index (OSI) to analyze the impact of the changing rate of BP.

**Result:**

The stresses at diastole were 2.5% ± 1.8% lower than that at systole under the same pressure drop during one cycle. It was also found that elevated SBP might cause the immediate increment of stress in the present cycle (292% ± 72.3%), but slight reduction in the successive cycle (0.48% ± 0.4%).

**Conclusion:**

The stress/strain distribution over the plaque is sensitive to the BP variability during one cardiac cycle, and the beat-to-beat BP variability could cause considerable impact on the progression of atherosclerosis in long-term.

## Introduction

Cardiovascular disease (CVD) has become more prevalent in the last decade. It is considered to be the major reason for the morbidity and mortality around the world [1]. Efforts have been made for better understanding of the causes and progression of CVD for early diagnosis of the risk of CVD. Especially, the atherosclerotic plaque leads to the reduction of artery lumen volume and the hardening of the vessel wall that would limit the blood supply. Insufficient blood supply is corresponding to the ischemia syndrome like dizziness and angina pectoris [2]. Moreover, about 70% of myocardial infarctions and sudden coronary deaths are the result of thrombosis from plaque rupture [3]. Early evaluation of plaque vulnerability is an effective way to screen the risk of these fatal events. Risk assessment of carotid atherosclerotic plaques is performed by evaluating the degree of luminal stenosis through imaging modalities including intra-vascular ultrasound (IVUS), Ultra-Sound Echo-Color-Doppler (US-ECD), magnetic resonance angiography(MRA), or computed tomography angiography(CTA) [4]. In addition, flow motion had been examined by means of medical imaging [5, 6], providing non-invasive assessment of hemodynamics over the plaque. But imaging-based assessment of vulnerable plaque tends to underestimate the risk of significant clinical events since the plaque vulnerability is not only depending on the morphology and distribution but also the composition of the plaque [7]. Therefore, the mechanical characteristics of plaque rupture have been studied by a number of experimental and numerical works [8–13].

Blood pressure is a well-known screening factor of CVD [14]. Realistic BP constantly varies during the cycle and fluctuates through beats [15]. A 3-year follow-up study conducted by Dirk, et al. concluded that high daytime systolic BP variability (over 15 mmHg) would increase the risk of early atherosclerosis development [16]. Iwata, et al. [17] had also come up with the similar conclusion from the follow-up study that day-by-day BP variability was associated with complex plaque in patient with severe aortic stenosis. Another followed-up study conducted by Nagai, et al. [18] reported that SBP fluctuations present a significant correlation with high risk CVD in the elderly that can serve as an indicator for carotid artery atherosclerosis. Meanwhile, heart rate had little to do with the variation in delta systolic blood pressure [19]. On the other hand, previous computational mechanical analyses had provided a strong correlation between blood flow/pressure and stress distribution over the plaque [10, 20–23]. Li, et al. [24] studied on how the structural factors (the thin fibrous cap, luminal stenotic degree, etc.) affect the stress distribution in the plaque. Tang, et al. [20] had included the atherosclerotic plaque volume, cap thickness, material properties, stenosis severity, asymmetry, etc. to analyze the combined effect to the plaque vulnerability. The flow patterns also significantly contribute to the plaque vulnerability. According to the literatures [25–27], the boundary conditions of inlet and outlet were either set with pulsatile pressure to analyze the effect from the maximum pressure drops, or pulsatile pressure inflow with constant pressure outflow to analyze the geometric effects. It was obvious that the magnitude of stresses varies with the BP variability, but further study of the effect caused by the varying ΔP was not examined yet.

In the present study, we will explore the influence of BP pattern on the plaque. We analyzed the effect of the maximum magnitude of SBP using computational mechanical analysis with one geometric model and pulsatile pressure input and output are implemented. The thickness of the fibrous cap is also taken account of. In addition, the effect of BP variability during the systole and diastole is compared, and beat-to-beat BP variability is included for the throughout understanding.

## Methods

### Geometric models

**Figure 1:**
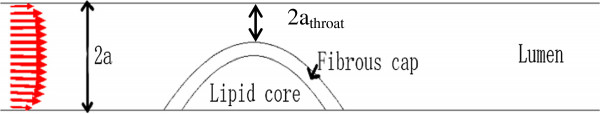
**The ideal carotid artery model with plaque structure, the plaque structure is composed of fibrous cap and lipid core.**

The geometry of the stenosed artery model is provided in Figure 1. It represents the cross-section of the carotid artery along the long axis where a solitary eccentric atherosclerotic plaque is sited. The shape of the plaque is modeled using the sinusoidal functions according to the previous study [24]:

12

where **a** and **a**_**throat**_ represent the radius of the lumen and the radius of the throat of the stenosis, respectively. **d** is the thickness of the fibrous cap. To simplify the ideal plaque model, the fibrous cap thickness is assumed homogeneously covering the lipid core. The stenosis severity defined as S equals to the ratio between height of the plaque and diameter of the healthy vessel [27]: 3

The same stenosis severity S equals to 62.5% is applied to all cases. The lumen radius of the model is **4 mm** and the plaque length is 20 mm for all cases.

The length of artery model is 100 mm. The plaque structure is set up 32 mm away from the inlet boundary which is necessary to the establishment of the laminar flow condition, 48 mm from the outlet boundary that which is considered sufficient for the establishment of flow recirculation at downstream of the stenosis [28].

Fibrous cap thickness has been proved as a critical geometric characteristic for the vulnerability assessment of the plaque [29–31]. Three groups with different d values are studied separately (d = 0.2 mm, 0.5 mm, 1 mm).

### Numerical implementation

For the fluid part, the blood flow is assumed incompressible, laminar and Newtonian in the model. The governing equation for the simulation of blood flow uses incompressible Navier–Stokes equations with arbitrary Lagrangian–Eulerian (ALE) transformation [32]. This equation is suitable for the problems with fluid–structure interactions and frequent mesh adjustments. No-slip boundary is assumed at the vessel wall. The inflow is implemented with the pulsatile pressure waveform generated according to the experimental data from the literature [33]. 4567

where, ρ, is the density, u is the blood flow velocity, p is the pressure, and *μ* is the blood viscosity. F is body force, which is set to be NULL in our problem. V represents mesh velocity depicting the coupled fluid–structure interface interactions. The values of *ρ* and *μ* were taken to be 1050 kg/m^3^ and 0.0034N ⋅ s/m^2^, respectively [23].

For the plaque components, the fibrous cap and the lipid core are assumed to be hyper-elastic material and described by the Mooney-Rivlin model. The strain energy function was given as follows, 89

where *C*_*i*_ are model parameters, and *κ* is the bulk modulus. **I**_1_ and **I**_2_ are the strain invariants, **τ** is the right Cauchy Green deformation tensor [34]. In this study, the values are from the literature [16], for the fibrous cap: **C**_10_ = 9200 Pa, **C**_01_ = 0, bulk modulus **κ** = 3000 MPa, density **ρ** = ; for the lipid core: **C**_10_ = 500 Pa, **C**_01_ = 0, bulk modulus **κ** = 200 MPa, density **ρ** = 900 kg/m^3^.

**Figure 2:**
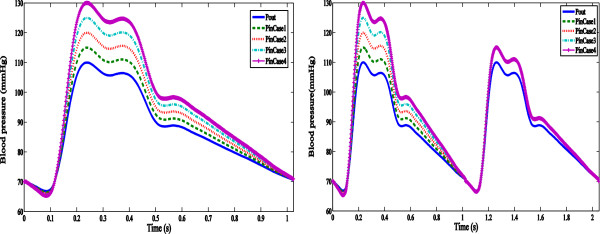
**The pressure waveforms for the inter-comparison set (left) and chain-comparison set (right) with four cases in each set.**

Since the pressure variation effects to the plaque is the key concern in this study, several inflow pressure waveforms had been generated to investigate the variation effect to the plaque by modifying the maximum SBP value [32]. Two sets were included: inter-comparison and chain comparison. For inter-comparison set, 4 comparing cases were generated. Maximum SBP increased by 5 mmHg in all groups of the inflow pressure waveform. For the chain-comparison set, another 4 comparing cases were generated; the pressure waveform was similar to that of the inter-comparison set in each case for the first cycle followed by the second cycle of which the maximum SBP was the same. The interval of 5 mmHg and the maximum difference of 20 mmHg were used according to the report from Sander et al. [16] as the pressure variation (below or above 15 mmHg) presented significant impact to the progression of atherosclerosis plaque. The outflow pressure waveforms were assumed the same in all cases. The pressure waveforms are illustrated in Figure 2. In combination of the sets of input boundary conditions and groups of geometric variations in the fibrous cap thickness, 24 simulations in total were performed in this study (Table 1).

**Table 1:** **This study including two sets, with 4 cases in each set and 3 groups in each case**

Sets	Group of thickness (Unit: mm)	d = 0.2		d = 0.5		d = 1	
Inter-comparison	Case1_1	^△^P =5		^△^P =5		^△^P =5	
	Case2_1	^△^P =10		^△^P =10		^△^P =10	
	Case3_1	^△^P =15		^△^P =15		^△^P =15	
	Case4_1	^△^P =20		^△^P =20		^△^P =20	
Chain-comparison	Case1_2	^△^P =5	^△^P =5	^△^P =5	^△^P =5	^△^P =5	^△^P =5
	Case2_2	^△^P =10	^△^P =5	^△^P =10	^△^P =5	^△^P =10	^△^P =5
	Case3_2	^△^P =15	^△^P =5	^△^P =15	^△^P =5	^△^P =15	^△^P =5
	Case4_2	^△^P =20	^△^P =5	^△^P =20	^△^P =5	^△^P =20	^△^P =5

The coupled fluid–structure interaction simulation in our study was solved by commercial finite-element software COMSOL that has been used and validated by previous studies [22, 24, 35]. It is appropriate for simulating the coupled physical processes described via partial differential equations. Detail of the modeling code and theoretical information can be found in the Modeling Guide (Comsol Multiphysics).

## Results

The present study is to investigate how the varying continuous BP and **△**P effect on the stress distribution and hemodynamic in the stenotic artery, wall shear stress (WSS), shear stress (SS) and the von Mises stress (VMS) were analyzed. To clarify the WSS vector deflection from blood flow predominant direction, the oscillation shear index (OSI) was calculated [36]: 10

**Figure 3:**
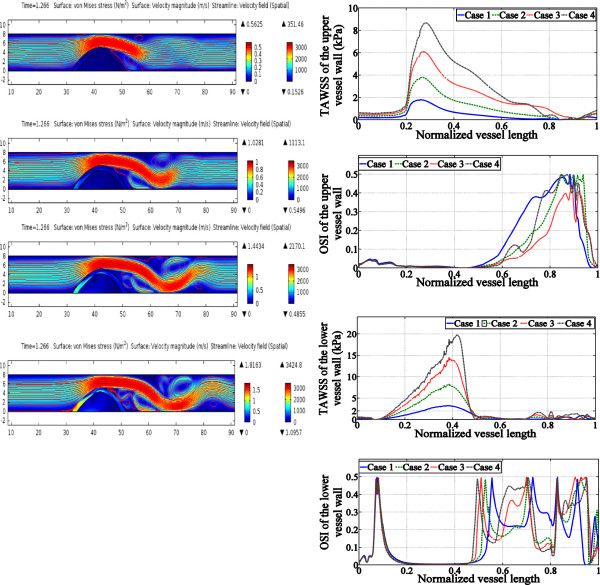
**Stress/strain distribution and the flow distribution of 4 cases in the inter-comparison set (left column from the top to bottom in the sequence of Case 1 to Case 4, the horizontal axis indicates vessel length and the perpendicular axis indicates vessel diameter, unit is mm.** The left color bar indicates velocity magnitude, unit is m/s and the right one indicates stress magnitude, unit is Pa.) OSI of upper wall shows oscillation at distal downstream with low WSS according to the TAWSS distribution, and excessive oscillation is found in the immediate downstream with low shear in all cases.

As the increase of the maximum SBP, being the engine applied to the blood flow increases. As a result, there were changes in the fluid dynamic and stress environment. The flow velocity and the stress distribution in the vessel of 62.5% stenosis are illustrated in Figure 3. Disturbance of the flow was found around the stenosis. Recirculation zone was generated along the vessel wall with the plaque and flow separation appeared along the healthy side. The flow field of the recirculation areas became more complex in larger SBP condition, and the recovery of the flow appeared to be extended further away from the stenosis. Stress and strain distribution mainly concentrated on the upstream wall of the plaque close to the border between the plaque and the healthy vessel wall. Time average WSS (TAWSS) under increasing ^△^P shared the similar distribution pattern along the walls with the increasing magnitude accordingly. Along the healthy side of the wall, the area of high OSI was larger in the cases with the highest and lowest ^△^P than that in the cases with ^△^P in between. In the contrary, the complexity of the OSI distribution along the plaque side wall was similar in all cases, but a larger area of high OSI can be found with increasing ^△^P.

**Figure 4:**
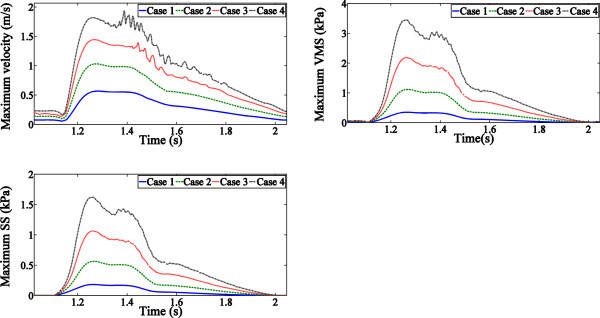
**Variation of the parameters during the cycle showed similar pattern to the pressure waveform in each case.** No significant difference of pattern is found among groups.

**Figure 5:**
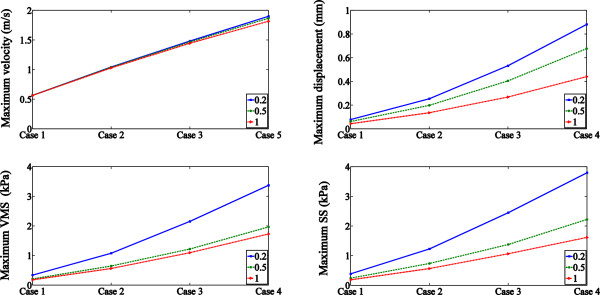
**Maximum value of parameter of groups are illustrated.** Thin fibrous cap significantly magnified the effect of the varied pressure to the stress distribution but the limited displacements of the plaque lead to the slight increments in the value of maximum velocity.

Figure 4 shows the effect of varied ^△^P to the maximum flow velocity profile and the maximum stress/strain magnitudes. The magnitude curves share the similar pattern of the pressure waveforms. Maximum flow velocity varied under different maximum ^△^P as well as the stress/strain in the fibrous cap and the lipid core. It was observed that the increase of maximum ^△^P led to monotonicity ascending in the velocity and the stress/strain. The maximum velocity increased by 82.8% (Case 2), 157.8% (Case 3) and 247.7% (Case 4) compare to Case 1, respectively. The VMS increased by 216.1% (Case 2), 515.7% (Case 3) and 875.8% (Case 4) compare to the Case 1, respectively. Displacement of the plaque was minor, but it did contribute to the difference in the maximum velocity under high SBP. The difference between groups is showed in Figure 5.

### Effect of different ^**Δ**^P

For the inter-comparison set, we introduce two phases into the inflow pressure waveform, the phases I indicates the period of pressure ascending from last diastolic pressure to the peak systolic pressure, the phase II indicates the period of pressure descending from the second peak pressure value (the dicrotic pulse) to the end diastolic pressure. The ^△^P values were selected by finding the same ^△^P value in both phases so that the stress and velocity were affected only by different pressure changing rate. We noticed that, magnitudes of stresses were higher in the phase I, while the magnitudes of velocities were generally larger in phase II except it was almost even in the cases with the thin fibrous cap (0.2) and high P. Detail values are illustrated in Table 2.

**Table 2:** **Detail data of the results show differences in the parameters under the same** ^**Δ**^ **P**

^Δ^ P (mmHg)	Velocity (m/s)		SS (kPa)		von Mises stress (kPa)	
1 mm	Phase I	Phase II	Phase I	Phase II	Phase I	Phase II
17.59	1.599	1.629	1.35	1.287	2.772	2.708
12.75	1.233	1.299	0.8612	0.835	1.715	1.719
8.3	0.8552	0.9446	0.4542	0.4515	0.8765	0.907
4	0.4468	0.5331	0.1519	0.1494	0.2868	0.2944
**Group a. The thickness of the fibrous cap equals to 1 mm**						
^Δ^ **P (mmHg)**	**Velocity (m/s)**		**SS (kPa)**		**von Mises stress (kPa)**	
**0.5 mm**	**Phase I**	**Phase II**	**Phase I**	**Phase II**	**Phase I**	**Phase II**
17.59	1.646	1.674	1.842	1.631	3.199	2.884
12.75	1.262	1.341	1.126	1.086	1.972	1.906
8.3	0.8623	0.9496	0.5907	0.5859	1.030	1.023
4	0.4482	0.5361	0.1969	0.1951	0.3424	0.3396
**Group b. The thickness of the fibrous cap equals to 0.5 mm**						
^Δ^ **P (mmHg)**	**Velocity (m/s)**		**SS (kPa)**		**von Mises stress (kPa)**	
**0.2 mm**	**Phase I**	**Phase II**	**Phase I**	**Phase II**	**Phase I**	**Phase II**
17.59	1.672	1.671	3.286	2.984	5.702	5.179
12.75	1.276	1.342	1.979	1.853	3.440	3.222
8.3	0.8655	0.9522	0.989	0.941	1.723	1.671
4	0.4481	0.5368	0.324	0.3055	0.566	0.5342
**Group c. The thickness of the fibrous cap equals to 0.2 mm**						

(a, b and c indicate different groups, respectively).

### Chain-reacting superimposing effect of different ^Δ^P

The pressure is fluctuating throughout the day and so does the blood flow. The fluctuation is normally placed, but the variation could lead to unexpected clinical events. There was no significant difference in flow distribution and the stress/strain on the plaque between the first cycles in the chain-comparison set and the value of the inter-comparison set. For the second cycle, the waveform pattern matched well in all cases compare to the Case 1. Still, minor difference can be found in the maximum values during the second cycle (Table 3). However, the maximum flow rate reduced by 48.95% (Case2), 69.14% (Case 3) and 73.58% (Case 4) in association with the pressure drops, respectively. The declining flow rate is a direct indication of blood supply reduction.

**Table 3:** **Detail results of the second cycle show minor difference in the parameters**

^Δ^ P (mmHg)	Max velocity (m/s)			Max SS (kPa)			Max VMS (kPa)		
	0.2	0.5	1	0.2	0.5	1	0.2	0.5	1
5	0.5699	0.5693	0.5675	0.3851	0.2392	0.1825	0.6728	0.416	0.3514
10	0.5695	0.5688	0.5671	0.3848	0.2386	0.1823	0.6722	0.4151	0.3512
15	0.5691	0.5686	0.5653	0.3824	0.2384	0.1808	0.668	0.4146	0.3491
20	0.5697	0.5673	0.567	0.3825	0.2369	0.182	0.6683	0.4121	0.3512

## Discussion

The pressure variations and the hemodynamic characteristics of the blood flow field contribute to the localization and the progression of atherosclerosis in the vessel. Besides the absolute BP value, clinical trials had indicated that BP variability was accounted partly for the outcomes of adverse cardiovascular consequences [37]. For better understanding of the biomechanical environment induced by the varied pressure, we simulated hemodynamics in an artery model with stenosis under the condition of pulsatile pressure, and the physiological parameters of specific pressure drops are analyzed. By comparing the hemodynamic distribution and the structural parameters, we had quantified the impact from BP variability to the vessel with a narrowed lumen, providing the comprehensive analyses of the potential risk of damage. Previous studies showed that the cap thickness acted as one primary factor for the stress distribution in the plaque [29, 38–40]. The results of our work also showed consistency to the conclusion. Variety pressure drops effect on the stress distribution in the plaque can be seen from the literatures [27, 41]. However, we provide a further investigation to the effect of the variety ^△^P during one cycle and the ^△^P over a couple cycles on the stress/strain distribution on the plaque. The results of our study showed that the varied pressure could result in different impact at the same ^△^P.

As it is shown in Figures 5, the maximum magnitude of the velocity and the stress is highly related to the ^△^P, which is the direct response to the driving force from the pressure drops. The thinner fibrous cap also contributed to the increase of stress magnitude and it was more significant under higher ^△^P. The pressure waveform used in this study consisted of three parts. The phase I and phase II as have been mentioned before, and the phase of the dicrotic pulse. The dicrotic pulse is believed to be the result of the vessel compliance. The lumen volume expands during the systole of the cardiac cycle reserving extract volume of blood, and then the volume of blood is re-ejected to the peripheral during the diastole of the cardiac cycle causing the second peak in the BP/velocity waveform. Compliance of the vessel is one important cardiovascular risk factor, and is usually measured by ultrasound as a pressure (carotid artery) and volume (outflow into the aorta) relationship [42]. When the ^△^P is lower (5 mmHg to 15 mmHg), the dicrotic phase can be found in the physiology parameter waveforms contributed to the plateau of the post-systolic period, and the significant difference contributed to the second peak magnitude after the systolic pulse at high ^△^P (20 mmHg). This finding could imply the measurement of blood flow velocity in stenotic artery by ultrasound devices can still provide, not rigorously, information about whether the elasticity of the central artery has been compromised due to pathological issues. Clinical statistics reported that the identifying of atherosclerosis in the carotid artery showed a correlation to the pathological changing in the central arteries [43–46]. Our results also implied that the measurement from peripheral hardening vessel segment for the evaluation to the vessel condition in the central can rely on the velocity waveform under the condition of high pressure (For example, after exercise), besides the morphology and intima-median thickness (IMT) of the vessel wall.

Stress and strain of the plaque are obviously influenced by ^△^P, the increasing trend of the magnitudes in Figure 4 is suggesting that increasing ^△^P could induce greater impact at the plaque. On the other hand, stress in the plaque was larger in the systolic period than that during diastole under the same ^△^P. We figured the reason for this phenomenon is laid on the pressure gradient as the slope was greater at systole than that at diastole.

Wall shear stress at the vessel wall acts as an important role in the remodeling of the intima. In Figure 3, higher ^△^P resulted in higher velocity at the throat as well as at the immediate downstream. However, viscosity, a physiological characteristic of blood, contributed to the flow separation phenomena at the downstream. Figure 3 presents the time average WSS (TAWSS) distribution during the cycle under variety ^△^P. The lengths of the low and oscillate WSS zones (<1.5 Pa (15 dyne/cm^2^) [47]) were similar at the lower wall under different ^△^P according to the OSI distributions, except when ^△^P was at 5 mmHg that low WSS could be found along the whole vessel wall. Additionally, the vertex region along the upper wall under higher ^△^P was found extended towards to the lower vessel wall. Atherosclerosis is not a stable disease, multiple factors will contribute to the progression of the plaque, but initiation is mainly due to pathological changes of endothelial cell caused by the WSS changes that initiating lesion on the intima [48–51]. Our result could imply that the varied pressure is not only positively correlated to the enlargement of plaque burden of original plaque, but also the initiation of the new lesion at the downstream which leads to the finding of complicated plaque distribution in clinical examinations. Especially, when the ^△^P is higher, substances in the blood were more likely to recirculate to the low WSS region, potentially increase the risk of plaque growth.

In this study, we quantified the stress/strain variation under variety ^△^P condition numerically. However, the pressure variation is different from person to person, time to time. Limitations of this study mainly come down to two aspects: the patient specific image-base geometry and the real time physiological parameters measuring. The geometry of the vessel and atherosclerotic plaque were ideal, for better accuracy, image-base geometry and vessel wall components could be adopted into the model. The pressure waveform used in this study is based on the previous works, yet, in the purpose of evaluating plaque progression, the analysis should rely on the real-time patient specific data because the realistic blood flow field is involving complicated interactions of multiple parameters. Vessel wall is assumed rigid in this study. Material properties of the plaque components are based on the literatures, and the nature of elasticity of the tissue would alter the actual stress/strain distribution over the plaque.

## Conclusion

In this study, with the use of computer simulation tool, we presented an extended analysis of the pressure variation impact on the vulnerability of the atherosclerosis plaque. With the advancing technique in the ultrasound imaging, we believe that far more information can be achieved through the clinical practice. Therefore, the 2-dimentional image-based CVD risk assessment is still valuable. We calculated the stress/strain variation and the blood flow field distribution in idealized 2-dimentional models. The results showed that high pressure drop not only increased the plaque vulnerability, but also creating a pathological WSS environment for the further growth of the plaque on the healthy side of the vessel wall. The plaque cap thickness could amplify the impact from the pressure variation. Although the beat-to-beat BP variability did not present a dramatic impact to the stability of the plaque instantly in this study, there is a considerable accumulation in the long run which is consistence to the chronic nature of the progression of atherosclerosis. In general, our study had presented the potential of further information achieved from pressure variation for risk assessment of CVD. For more accurate evaluation of plaque vulnerability, further study will be conducted to address the limitation mentioned above.

## Abbreviations

BP: Blood pressure
τ: Right Cauchy green tensor
F: Body force
SS: Shear stress in the plaque
κ: Bulk modulus (Pa)
W: Stain energy function
ρ: Density (kg/m^3^)
SBP: Systolic blood pressure
C10 C01: Material parameters (Pa)
DBP: Diastolic blood pressure
V: Mesh velocity
d: Thickness of the fibrous cap (mm)
P: Pressure (mmHg)
t: Time (s)
^△^P: Pressure difference (mmHg)
u: Velocity (m/s) of blood
a: Radius of the vessel (mm)
μ: Viscosity of blood (Pa•s)
CVD: Cardiovascular disease
VMS: Von Mises stress in the plaque (kPa)
OSI: Oscillation index of WSS
WSS: Wall Shear Stress on the vessel wall (Pa)
IMT: Intima-media thickness
TAWSS: Time averaged WSS

## References

[1] Mathers CD, Fat DM, Boerma J (2008). The global burden of disease: 2004 update.

[2] AbuRahma AF, Bandyk DF (2013). Noninvasive vascular diagnosis: a practical guide to
therapy.

[3] Naghavi M, Libby P, Falk E, Casscells SW, Litovsky S, Rumberger J, Badimon JJ, Stefanadis C, Moreno P, Pasterkamp G, Fayad Z, Stone PH, Waxman S, Raggi P, Madjid M, Zarrabi A, Burke A, Yuan C, Fitzgerald PJ, Siscovick DS, de Korte CL, Aikawa M, Airaksinen KE, Assmann G, Becker CR, Chesebro JH, Farb A, Galis ZS, Jackson C, Jang IK (2003). From vulnerable plaque to vulnerable patient: a call
for new definitions and risk assessment strategies: Part II. Circulation.

[4] Græbe M, Sillesen H, Kjær A, Højgaard L (2011). Carotid plaque imaging with FDG-PET and
ultrasound. Imag Med.

[5] Wong KKL, Kelso RM, Worthley SG, Sanders P, Mazumdar J, Abbott D (2009). Noninvasive cardiac flow assessment using high speed
magnetic resonance fluid motion tracking. PLoS ONE.

[6] Wong KKL, Kelso RM, Worthley SG, Sanders P, Mazumdar J, Abbott D (2009). Theory and validation of magnetic resonance fluid
motion estimation using intensity flow data. PLoS ONE.

[7] Falk E (1992). Why do plaques rupture?. Circulation.

[8] Li ZY, Taviani V, Tang T, Sadat U, Young V, Patterson A, Graves M, Gillard JH (2009). The mechanical triggers of plaque rupture: shear
stress vs pressure gradient. Br J Radiol.

[9] Tang D, Teng Z, Canton G, Yang C, Ferguson M, Huang X, Zheng J, Woodard PK, Yuan C (2009). Sites of rupture in human atherosclerotic carotid
plaques are associated with high structural stresses: an in vivo MRI-based 3D
fluid-structure interaction study. Stroke; a journal of cerebral circulation.

[10] Huang Y, Teng Z, Sadat U, He J, Graves MJ, Gillard JH (2013). In vivo MRI-based simulation of fatigue process: a
possible trigger for human carotid atherosclerotic plaque rupture. BioMedical Engineering Online.

[11] Hatsukami TS, Ross R, Polissar NL, Yuan C (2000). Visualization of fibrous cap thickness and rupture in
human atherosclerotic carotid plaque in vivo with high-resolution magnetic
resonance imaging. Circulation.

[12] Yang C, Bach RG, Zheng J, Ei Naqa I, Woodard PK, Teng Z, Billiar K, Tang D (2009). In vivo IVUS-based 3-D fluid–structure interaction
models with cyclic bending and anisotropic vessel properties for human
atherosclerotic coronary plaque mechanical analysis. Biomedical Engineering, IEEE Transactions on.

[13] Wong KKL, Tu JY, Mazumdar J, Abbott D (2010). Modelling of blood flow resistance for an
atherosclerotic artery with multiple stenoses and poststenotic
dilatations. ANZIAM Journal E.

[14] Kannel WB (1996). Blood pressure as a cardiovascular risk factor:
prevention and treatment. JAMA.

[15] Yano Y, Kario K (2012). Nocturnal blood pressure, morning blood pressure
surge, and cerebrovascular events. Curr Hypertens Rep.

[16] Sander D, Kukla C, Klingelhöfer J, Winbeck K, Conrad B (2000). Relationship between circadian blood pressure patterns
and progression of early carotid atherosclerosis A 3-year follow-up
study. Circulation.

[17] Iwata S, Sugioka K, Matsumura Y, Fujita S, Ito A, Hozumi T, Hanatani A, Yoshiyama M (2013). Relationship between day-by-day blood pressure
variability and aortic arch atherosclerosis. Eur Heart J.

[18] Nagai M, Hoshide S, Ishikawa J, Shimada K, Kario K (2011). Visit-to-visit blood pressure variations: new
independent determinants for carotid artery measures in the elderly at high risk
of cardiovascular disease. J Am Soc Hypertens.

[19] Nagai M, Hoshide S, Kario K (2011). Visit-to-visit blood pressure variability and carotid
artery atherosclerosis: heart rate was not a confounder. Hypertension.

[20] Tang D, Yang C, Zheng J, Woodard PK, Saffitz JE, Petruccelli JD, Sicard GA, Yuan C (2005). Local maximal stress hypothesis and computational
plaque vulnerability index for atherosclerotic plaque assessment. Ann Biomed Eng.

[21] Kock SA, Nygaard JV, Eldrup N, Frund ET, Klaerke A, Paaske WP, Falk E, Yong Kim W (2008). Mechanical stresses in carotid plaques using MRI-based
fluid–structure interaction models. J Biomech.

[22] Lorenzini G, Casalena E (2008). CFD analysis of pulsatile blood flow in an
atherosclerotic human artery with eccentric plaques. J Biomech.

[23] Melih Guleren K (2013). Numerical flow analysis of coronary arteries through
concentric and eccentric stenosed geometries. J Biomech.

[24] Li ZY, Howarth SP, Tang T, Gillard JH (2006). How critical is fibrous cap thickness to carotid
plaque stability? A flow-plaque interaction model. Stroke.

[25] Tang D, Yang C, Zheng J, Woodard PK, Saffitz JE, Sicard GA, Pilgram TK, Yuan C (2005). Quantifying Effects of Plaque Structure and Material
Properties on Stress Distributions in Human Atherosclerotic Plaques Using 3D FSI
Models. J Biomech Eng.

[26] Tang D, Yang C, Ku DN (1999). A 3-D thin-wall model with fluid–structure
interactions for blood flow in carotid arteries with symmetric and asymmetric
stenoses. Comput Struct.

[27] Tang D, Yang C, Kobayashi S, Zheng J, Vito RP (2003). Effect of stenosis asymmetry on blood flow and artery
compression: a three-dimensional fluid–structure interaction
model. Ann Biomed Eng.

[28] Belzacq T, Avril S, Leriche E, Delache A (2012). A numerical parametric study of the mechanical action
of pulsatile blood flow onto axisymmetric stenosed arteries. Med Eng Phys.

[29] Wenk JF (2011). Numerical modeling of stress in stenotic arteries with
microcalcifications: a parameter sensitivity study. J Biomech Eng.

[30] Gao H, Long Q, Kumar Das S, Halls J, Graves M, Gillard JH, Li ZY (2011). Study of carotid arterial plaque stress for
symptomatic and asymptomatic patients. J Biomech.

[31] Wenk JF, Papadopoulos P, Zohdi TI (2010). Numerical modeling of stress in stenotic arteries with
microcalcifications: a micromechanical approximation. J Biomech Eng.

[32] Pu L, Xiong H, Liu X, Zhang H, Zhang Y-T (2014). Quantifying Effect of Blood Pressure on Stress
Distribution in Atherosclerotic Plaque. The International Conference on Health Informatics, IFMBE
Proceedings.

[33] Yang C, Tang D, Yuan C, Hatsukami TS, Zheng J, Woodard PK (2007). In vivo/ex vivo MRI-based 3D non-Newtonian FSI models
for human atherosclerotic plaques compared with fluid/wall-only
models. Comput Model Eng Sci.

[34] Bathe K-J (2006). Finite element procedures.

[35] Liu B (2005). Computer simulations of flows in curved tubes with
stenosis. Proceedings of the COMSOL Multiphysics User's Conference.

[36] Gao H, Long Q, Sadat U, Graves M, Gillard JH, Li ZY (2009). Stress analysis of carotid atheroma in a transient
ischaemic attack patient using the MRI-based fluid–structure interaction
method. Br J Radiol.

[37] Parati G, Ochoa JE, Lombardi C, Bilo G (2013). Assessment and management of blood-pressure
variability. Nat Rev Cardiol.

[38] Koivistoinen T, Virtanen M, Hutri-Kahonen N, Lehtimaki T, Jula A, Juonala M, Moilanen L, Aatola H, Hyttinen J, Viikari JS, Raitakari OT, Kahonen M (2012). Arterial pulse wave velocity in relation to carotid
intima-media thickness, brachial flow-mediated dilation and carotid artery
distensibility: the Cardiovascular Risk in Young Finns Study and the Health 2000
Survey. Atherosclerosis.

[39] Nakazawa G, Yazdani SK, Finn AV, Vorpahl M, Kolodgie FD, Virmani R (2010). Pathological findings at bifurcation lesions: the
impact of flow distribution on atherosclerosis and arterial healing after stent
implantation. J Am Coll Cardiol.

[40] Gao H, Long Q (2008). Effects of varied lipid core volume and fibrous cap
thickness on stress distribution in carotid arterial plaques. J Biomech.

[41] Tang D, Yang C, Kobayashi S, Ku DN (2001). Steady flow and wall compression in stenotic arteries:
a three-dimensional thick-wall model with fluid–wall interactions. J Biomech Eng.

[42] Nestel PJ, Pomeroy S, Kay S, Komesaroff P, Behrsing J, Cameron JD, West L (1999). Isoflavones from Red clover improve systemic arterial
compliance but Not plasma lipids in menopausal women 1. J Clin Endocrinol Metabol.

[43] Craven T, Ryu J, Espeland M, Kahl F, McKinney W, Toole J, McMahan M, Thompson C, Heiss G, Crouse J (1990). Evaluation of the associations between carotid artery
atherosclerosis and coronary artery stenosis. A case–control
study. Circulation.

[44] Hodis HN, Mack WJ, LaBree L, Selzer RH, Liu C-r, Liu C-h, Azen SP (1998). The role of carotid arterial intima-media thickness in
predicting clinical coronary events. Ann Intern Med.

[45] Inaba Y, Chen JA, Bergmann SR (2012). Carotid plaque, compared with carotid intima-media
thickness, more accurately predicts coronary artery disease events: a
meta-analysis. Atherosclerosis.

[46] Salonen JT, Salonen R (1991). Ultrasonographically assessed carotid morphology and
the risk of coronary heart disease. Arterioscler Thromb Vasc Biol.

[47] Cheng C, Helderman F, Tempel D, Segers D, Hierck B, Poelmann R, van Tol A, Duncker DJ, Robbers-Visser D, Ursem NT (2007). Large variations in absolute wall shear stress levels
within one species and between species. Atherosclerosis.

[48] Miyazaki Y, Nomura S, Miyake T, Kagawa H, Kitada C, Taniguchi H, Komiyama Y, Fujimura Y, Ikeda Y, Fukuhara S (1996). High shear stress can initiate both platelet
aggregation and shedding of procoagulant containing
microparticles. Blood.

[49] Carallo C, Irace C, Pujia A, De Franceschi MS, Crescenzo A, Motti C, Cortese C, Mattioli PL, Gnasso A (1999). Evaluation of common carotid hemodynamic forces
Relations with wall thickening. Hypertension.

[50] Chatzizisis YS, Coskun AU, Jonas M, Edelman ER, Feldman CL, Stone PH (2007). Role of endothelial shear stress in the natural
history of coronary atherosclerosis and vascular remodelingmolecular, cellular,
and vascular behavior. J Am Coll Cardiol.

[51] Kroll MH, Hellums JD (1996). Platelets and Shear Stress. J Am Soc Hematol.

